# Semantic Segmentation Leveraging Simultaneous Depth Estimation

**DOI:** 10.3390/s21030690

**Published:** 2021-01-20

**Authors:** Wenbo Sun, Zhi Gao, Jinqiang Cui, Bharath Ramesh, Bin Zhang, Ziyao Li

**Affiliations:** 1 School of Remote Sensing and Information Engineering, Wuhan University, Wuhan 430079, China; bin.zhang@whu.edu.cn (B.Z.); liziyao@whu.edu.cn (Z.L.); 2 Peng Cheng Laboratory, Shenzhen 518055, China; cuijq@pcl.ac.cn; 3 The N.1 Institute for Health, National University of Singapore, Singapore 117411, Singapore; lsirame@nus.edu.sg

**Keywords:** CNN, semantic segmentation, depth estimation, multi-source feature fusion

## Abstract

Semantic segmentation is one of the most widely studied problems in computer vision communities, which makes a great contribution to a variety of applications. A lot of learning-based approaches, such as Convolutional Neural Network (CNN), have made a vast contribution to this problem. While rich context information of the input images can be learned from multi-scale receptive fields by convolutions with deep layers, traditional CNNs have great difficulty in learning the geometrical relationship and distribution of objects in the RGB image due to the lack of depth information, which may lead to an inferior segmentation quality. To solve this problem, we propose a method that improves segmentation quality with depth estimation on RGB images. Specifically, we estimate depth information on RGB images via a depth estimation network, and then feed the depth map into the CNN which is able to guide the semantic segmentation. Furthermore, in order to parse the depth map and RGB images simultaneously, we construct a multi-branch encoder–decoder network and fuse the RGB and depth features step by step. Extensive experimental evaluation on four baseline networks demonstrates that our proposed method can enhance the segmentation quality considerably and obtain better performance compared to other segmentation networks.

## 1. Introduction

Semantic segmentation aims at predicting a class label for each pixel in the image, which plays a crucial role in various applications, including autonomous driving [1,2,3], robotics [4,5], medical applications [6], and augmented reality [7]. Because of the success of CNN in recent years, a large number of semantic segmentation algorithms based on deep learning have been proposed, which have made a breakthrough in this filed [8,9,10,11].

**Figure 1:**
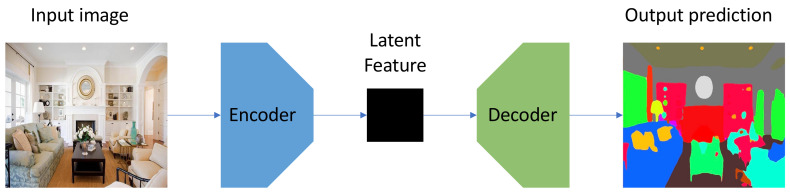
The architecture of a simple encoder–decoder model.

Deep learning techniques based on CNNs, which can naturally integrate the feature extraction and classification into an end-to-end manner, have made a vast contribution to semantic segmentation and obtained the state-of-the-art performance on benchmark datasets. Among the various CNN architectures, the encoder–decoder structure is widely used and can usually obtain excellent performance [12,13,14]. Figure 1 demonstrates the architecture of a simple encoder–decoder model. As the name suggests, the network consists of an encoder and a decoder. The former maps a high dimensional input (usually an image) to a lower dimensional feature space, while the latter can capture sharper boundaries of objects in the scene by gradually recovering the latent spatial information. Nevertheless, this traditional single branch architecture cannot process the multi-input (such as RGB and depth map) separately, making it difficult to extract a variety of context information. Adding extra branches to process multi-input is an intuitive idea to solve this problem [15,16]. However, there is still no experiment that investigates the performance on different combinations of branches that have different network architectures, let alone any general design that can flexibly combine different networks into an integral multi-branch architectures.

Images taken by monocular cameras only contain three color channels of RGB. When humans observe these 2D RGB images, we can easily reconstruct the location distribution and geometric relations of objects in the real scenes according to previous experience. However, for CNN, the input RGB images only reflect the color and texture information of the scenes, but do not record the location distribution and geometric relations of objects in the scenes. This is caused by the inability of CNN to directly obtain depth information from RGB images. In addition, reference [17] discovered that each segmentation class usually had similar depth and distribution in the scene. Most studies [18,19,20] focus on the segmentation on RGBD images which has the ground truth of depth information; however, for monocular RGB images, the optimal way to improve segmentation performance utilizing depth information has been left an open question. Therefore, we believe that the lack of depth information may lead to sub-optimal results on RGB images. Considering that, if we can obtain the depth information of RGB images, it will be an extra source for the network to improve the segmentation quality.

Based on the above observation, we propose a semantic segmentation network leveraging simultaneous depth estimation. Specifically, we utilize a depth estimation network [21] to obtain the depth information of RGB image datasets, and treat the depth map as one of the inputs to the network which will guide the network to obtain better segmentation performance. In addition, based on the traditional encoder–decoder structure, we add a new encoder branch to extract the underlying semantic features in the depth map. In order to fully learn the RGB and depth information, we proposed a feature fusion strategy to fuse the two kinds of the information step by step. Finally, the decoder restores the fused feature to the image resolution and outputs the prediction. In our experiments, we investigate the relationship between various combinations of RGB and depth encoders—including different backbone networks—on performance and obtain better performance compared to other representative segmentation networks.

In summary, our method can improve the segmentation quality by fusing the depth feature into RGB feature, even without the ground truth of the depth in the datasets. In addition, compared with the simple element-wise summation strategy proposed in [15], our method allows encoders with different structures to combine with each other and improves the segmentation performance.

The paper makes main contributions as follows:

- We propose a method to guide the RGB image semantic segmentation using depth information extracted from a depth estimation network.
- We propose a novel fusion strategy based on the multi-branch network architecture, which allows encoders with different structures to combine with each other and improves the segmentation performance.
- We train several networks using our proposed method on the ADE20k [22] dataset without any extra data source. Experiments show that our method can improve the segmentation performance compared with the baseline model.

The rest of this paper is organized as follows. Section 2 presents the related work on semantic segmentation networks and other research related to our work. Section 3 discusses our proposed method in detail. Section 4 elaborates the experiments and the quantitative and qualitative results based on several representative models. Finally, the conclusions and future works are discussed in Section 5.

## 2. Related Work

### 2.1. Semantic Segmentation Networks

Before deep learning was applied to computer vision tasks, most image segmentation methods were based on hand-crafted features, such as thresholding [23], region splitting, or merging [24,25]. Some other pioneer works also focus on semantic segmentation in the framework of MPEG-7 standard [26,27,28]. Some other techniques used retrainable neural networks also provide another solution for semantic segmentation [29,30,31]. More recent algorithms often optimize the metric of intra-region similarity and inter-region dissimilarity, such as mean shift [32] and graph based image segmentation [33]. In recent years, many semantic segmentation problems are solved with deep learning, which is more accurate and efficient compared to traditional methods. In this section, we review some representative research based on their main technical contributions.

Fully convolutional networks. Fully Convolutional Networks (FCN) [34] was the first CNN network proposed for semantic segmentation. FCN replaced the full connected layers with convolution layers in the original image classification network (such as VGG16 [35] and GoogLeNet [36]), enabling the network to process non-fixed size of input images. In addition, skip connection and bilinear interpolation were used to restore the low-resolution feature map to the original resolution. The model achieved state-of-the-art performance on Pascal VOC [37], NYUDv2 [38], and SIFT Flow [39]. However, FCN did not take into account the globe-level semantic context which led to inaccurate results in some cases. In order to overcome this limitation, ParseNet [40] modified the structure of FCN using the average feature of a layer to augment the features at each location and produce a smoother segmentation result than original FCN. As a pioneering work in semantic segmentation, the formulation of FCN was followed by many related research [41,42,43]. CNNs with graphical models. To overcome the drawback that traditional CNNs cannot capture global context information well, many works focus on combining CNN with probabilistic graphical models. Chen et al. [44] proposed a network based on the combination of CNNs and fully connected Conditional Random Fields (CRFs). They noted that the last layer of the CNN was not sufficiently localized for accurate object segmentation. To solve this problem, they utilize a fully connected CRF to integrate more global context information. Compared to previous networks, their model is able to localize the object boundaries more accurately. Lin et al. [45] combined a contextual deep CRFs with the deep network to capture “patch–patch” context (between image regions) and “patch-background” context. The results showed this formulation was able to integrate more contextual information into the features and achieved the state-of-the-art performance on Pascal VOC 2012. Other similar works combine probabilistic graphical models and CNN include [46,47,48].

Encoder–decoder based networks. Another popular segmentation architecture in semantic segmentation field is encoder–decoder based networks. Badrinarayanan et al. [12] proposed Segnet, a symmetric encoder—decoder structure for semantic segmentation. In Segnet, both the encoder and decoder consisted of the 13 convolution layers in the VGG16 network and unpooling layers were used as the upsampling strategy to restore the resolution of feature map. HRNet [14] is also a typical encoder–decoder network. Other than recovering high resolution features such as Segnet, it proposed a method to keep the high resolution of feature graph in the whole process of convolution. By gradually adding low-resolution feature map subnetworks to the main network of high-resolution feature graph in parallel, different networks realized multi-scale fusion and feature extraction. Other encoder–decoder networks [13,49,50] also obtain inspiring performance.

Dilated convolution. Networks using dilated convolution have been widely used for semantic segmentation. Compared with traditional convolution, dilated convolution can obtain a bigger receptive field without sacrificing spatial resolution. One of the most popular networks is Deeplab family. DeepLab [44,51,52,53] series networks first proposed dilated convolution to address the decreasing resolution in the network (caused by max-pooling and striding) and realized a multi-scale receptive field via Atrous Spatial Pyramid Pooling (ASPP), thus enabling a robust segmentation of objects with multi-scale input. In addition, dilated convolution has been applied in various occasions due to excellent performance and no extra computation [54,55,56].

Attention based networks. Attention mechanisms have been explored in computer vision over the past years, and some works also tried to build their networks based on attention mechanisms for semantic segmentation. Huang et al. [57] proposed a network using reverse attention mechanisms, namely Reverse Attention Network (RAN). The network tried to learn the opposite concept, for example, the network can learn what a cat is and what is not a cat simultaneously, which is able to enhance intra-class similarity and inter-class dissimilarity to some extent. Li et al. [58] proposed a Pyramid Attention Network for semantic segmentation whose biggest difference from previous works lied in the fact that the author uses FPA (Feature Pyramid Attention) and GAU (Global Attention Upsample) instead of ASPP structure to extract features. Similar works include [59,60,61,62].

Networks for RGBD images segmentation. Many works also focused on the segmentation of RGBD image data. In [15,63,64,65], a new branch was added to process the depth information and fused the depth features into RGB features. Another idea is to encode a depth map into an HHA image (Horizontal delineation, Height above ground, and norm Angle), and then input the RGB image and HHA image into two branch networks, respectively, finally adding the outputs of the two networks together. Authors in [66,67,68,69] attempted to utilize 3D convolution to solve the segmentation problem of RGBD images, but led to high computation and memory consumption. Aiming at the drawback of high computation and memory consumption, a method of depth-aware convolution and depth-aware average pooling was proposed [70], which can improve the segmentation performance and reduce the network computation without introducing any additional parameters.

### 2.2. Depth from a Single Image

How to obtain depth information of real scenes from a single input image has been a challenging problem in computer vision field. Existing methods can be categorized into supervised learning and unsupervised/self-supervised learning methods. Eigen [71] et al. first proposed the method of multi-scale depth network to predict the depth map. Fu [72] et al. increased the speed of network convergence based on ordinal regression but might fall into local optimal solution in monocular depth estimation. Considering that a local constraint calculated on a small neighborhood did not fully utilize the geometric structure information of the scene, Yin [73] et al. proposed a more stable geometric constraint from a global perspective, which can considerably improve the depth prediction accuracy. On the other hand, since the acquisition of ground-truth of depth information is resource intensive, many methods with unsupervised/self-supervised have been proposed. Godard et al. [74] proposed a self-supervised network, which uses a minimum reprojection loss to handle occlusions robustly. Zhou et al. [75] put forward an unsupervised learning framework that can jointly predict the depth map and the ego-motion from the monocular video. Vincent et al. [76] introduced geometric structure in the learning process by modeling the scene and the individual objects to improve the prediction performance of monocular videos. In addition, various methods [77,78,79,80] are proposed to improve the performance of unsupervised/self-supervised depth estimation. Other research aimed at solving the poor generalization performance on unseen scenes outside the training set of traditional monocular depth estimation networks. For example, Yin [21] constructed a large scale and high-diversity RGBD dataset, and learned affine-invariant depth on the diverse dataset, which ensured both high generalization and high quality geometric shapes of scenes.

In the works mentioned above, some of them focus on introducing various network structures (such as dilated convolution) to improve the segmentation performance, while the others achieve higher accuracy by preprocessing the input data (such as the HHA image). However, few studies have focused on how to utilize depth information to assist network segmentation on the RGB data sets. Inspired by these works, we propose a method to realize semantic segmentation by simultaneous depth estimation. Specifically, we first obtain the depth map of input RGB images by a depth estimation network, which reflects the geometric relationship and distribution of scene objects to guide the semantic segmentation. In addition, based on the traditional encoder–decoder structure, we add an extra branch to process the depth map generated by the depth estimation network. In order to incorporate the depth information into the semantic segmentation framework, we propose a fusion strategy to fuse the RGB and depth features step by step. Our proposed formulation can be applied on various encoder–decoder networks flexibly. The experimental results show that the method can effectively improve the segmentation performance of the backbone networks.

## 3. Method

In this section, we discuss the architecture of our semantic segmentation network in detail and then introduce the depth estimation network.

### 3.1. Segmentation Network Structure

**Figure 2:**
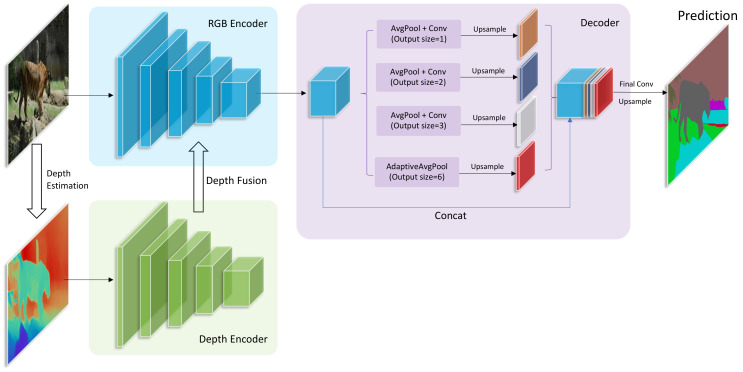
The detailed architecture of our proposed network. The inputs are an RGB image and its corresponding depth map. They are separately fed into two encoders. Then, depth features are fused into RGB features step by step. The fused features are fed into the decoder that consists of a PPM [81] module. At last, the decoder outputs the prediction of semantic segmentation.

We propose an encoder—decoder network structure, as shown in Figure 2. The network is mainly composed of two parts: (1) RGB encoder and depth encoder, the two encoders are respectively used to extract the feature map of RGB and depth input images; (2) Decoder, the decoder restores the low resolution feature map to the original size and predicts the output. We will discuss the structures in more detail below.

#### 3.1.1. RGB Encoder and Depth Encoder

As shown in Figure 2, we use the classic image classification networks as the backbone of the encoders (such as Resnet [82], VGG [35], and Mobilenetv2 [83]). After removing the final full connection layers and softmax layers, the model becomes a fully convolutional network, and we divide the model into several blocks at each down-sampling operation (such as pooling, striding convolution, and interpolation and so on). In each block of the model, the depth features are fused into the RGB encoder before the down-sampling operation (which will be discussed in detail below), and then the fused feature of this block is fed into the next block. Note that the structure of the original RGB encoder has not been changed at all, thus the final output feature has the same size with the original RGB encoder. This allows us to flexibly try a variety of combinations of RGB encoders and depth encoders. In this paper, we adopt DilatedResnet50, DilatedResnet101 DilatedMobileNetV2 which are proposed in [22], and HRNetV2 [14] as our RGB encoders. As for depth encoders, we adopt Resnet50 and VGG16. The performance of these combinations will be demonstrated in the experimental section.

#### 3.1.2. Fusion Strategies

**Figure 3:**
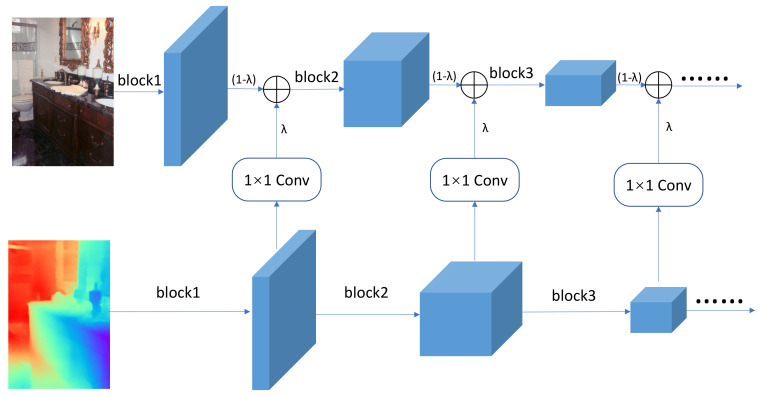
After each block of RGB encoder and depth encoder, the depth feature is fed into 1 × 1 convolution, and then we add the RGB and depth features together via a proportion parameter λ.

In this section, we will describe in detail how to fuse the output feature of each block in the depth encoder into the RGB encoder. Different from FuseNet [15] that simply uses an identical structure for both the RGB encoder and the depth encoder, our network adopts different architectures for them. Thus, an element-wise summation for RGB feature and depth feature without any processing no longer applies in this case. To allow the fusion process, we propose a novel fusion strategy that can eliminate the contradictions caused by different network structures and improve the performance by using properly the 1 × 1 convolution layers and a fused ratio coefficient. As shown in Figure 3, after each block of RGB and depth encoder, we add the output feature map element by element. We use two strategies to implement this operation.

On the one hand, because the RGB encoder and depth encoder have different architectures, their output feature size of each block may be different from each other. In order to add the two feature maps element by element, it is necessary to ensure that they have the same size. To ensure that the RGB and depth features have the same width and height, we only perform a feature fusion after each pooling layer or strided convolution where the output size will be halved. In addition, we utilize 1 × 1 convolution to ensure the depth feature map have the same number of channels as the RGB feature map. After that, the RGB and depth features have the same width, height, and number of channels, which enable us to add them element by element. Note that our fusion approach works only for the two encoders that use the same stride-2 operations.

On the other hand, to control the fusion proportion of RGB information and depth information, we introduce a coefficient λ, and use the following formula for fusion:

(1) $$f_{fusion}=\left(1-\lambda \right)f_{rgb}+\lambda f_{depth}$$

where $f_{fusion}$ denotes the feature map after fusion, $f_{rgb}$ denotes the RGB feature map, and $f_{depth}$ denotes the depth feature map. Changing the value of λ allows us to control the proportion of the two types of information. Specifically, the bigger λ is, the greater proportion of depth information in fusion features compared with RGB information, and vice versa. Considering the extreme case, when λ=0, depth information is not fused into RGB features, and the network degrades into the original RGB encoder–decoder structure. When λ=1, there is no RGB information in the fusion feature, and the network becomes a depth encoder–decoder structure. The value of λ indicates the proportion of depth features fused with RGB features, and is crucial to the segmentation performance of the network. In our experiments, we set the λ=1 to 0.4 for best performance.

#### 3.1.3. Decoder

**Figure 4:**
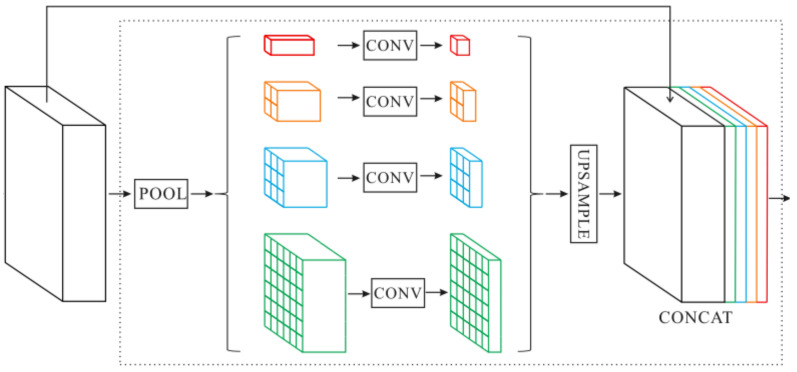
The architecture of the Pyramid Pooling Module (PPM).

In order to capture the different scales of global contextual information, we adopt the module proposed by [81], Pyramid Pooling Module (PPM), which is shown in Figure 4. In the segmentation of complex scenes, it is very important to obtain global contextual information from the input image. In the deep convolutional neural network, the size of the receptive field can roughly measure the amount of contextual information captured. In addition, the receptive field of the network calculated theoretically is potentially larger than the size of the input image. However, in fact, the empirical receptive field of the network can be smaller, especially in the high-level layers of the network [81]. The global average pooling can solve this problem well. We use four different scales of global average pooling layer (1×1,2×2,3×3,6×6) to process input features. After dimension reduction by 1×1 convolution layer, the low-resolution feature maps are restored to the input feature size by bilinear interpolation upsampling. Finally, we concatenate the features of different levels to obtain the final pyramid pooling global feature.

### 3.2. Depth Estimation Network

**Figure 5:**
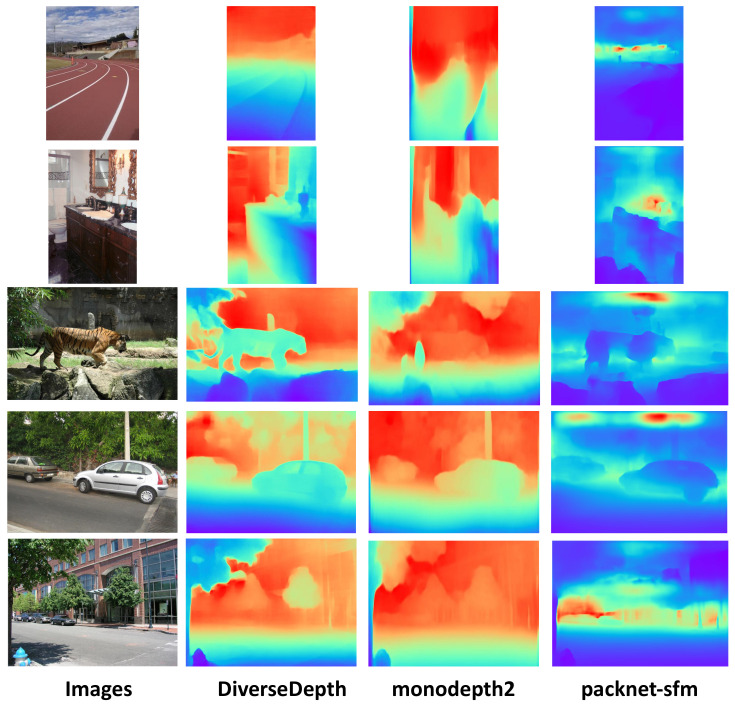
The comparison results of different depth estimation methods on the ADE20k dataset. Because of the lack of the ground truth, we do not retrain depth estimation models. The depth estimation results come from the application of the original models from [21,74,84], respectively. All of the images are not included in the training sets of these three models. The scene in the images includes indoor, outdoor, streetscape, and natural environment. Blue parts mean being closer to the camera, while red regions are farther.

In this paper, we try to estimate depth on RGB image datasets with no ground-truth information. In addition, an unsupervised depth estimation network usually requires camera calibration parameters or image data of previous and next frames in the video, which are difficult to obtain for RGB image datasets. In addition, in order to apply our model in various scenarios, it is important to choose a network that can well predict the depth of complicated scenes. Therefore, our method needs to select a depth estimation network with high generalization performance that can generate satisfactory results in various scenes to predict the depth information of RGB images. The work in [21] is able to meet our requirements. Different from KITTI, NYU, SUN-RGBD, and other datasets of low-diversity scenes, this paper constructed a dataset with tremendous diversity scenes by crawling stereo images or videos from the internet. In addition, the authors trained a model which utilized affine invariant to predict depth. Due to the use of the multi-curriculum learning policy, which sorts the training data by the increasing difficulty and samples a series of mini-batches that exhibit an increasing level of difficulty, the model trained on the constructed dataset has good generalization performance. Therefore, we use this model directly to predict the depth of the ADE20k dataset. To demonstrate the robustness of Diverse Depth, we also test the same images in the ADE20k dataset using other two monocular depth estimation methods, named monodepth2 [74] and packnet-sfm [84], respectively. As we can see in Figure 5, for images which are not included in their training datasets, monodepth2 and packnet-sfm can not estimate the depth well, and the boundary of different objects is fuzzy compared to the prediction of Diverse Depth. In contrast, Diverse Depth [21] performs well on the ADE20k dataset. In the predicted depth map, the boundary of objects is easy to distinguish, and the spatial layout of the image can be reflected to some extent which will be useful supplements for RGB information. As for the generalization, the performance of monodepth2 and packnet-sfm is mixed in various scenes, and, by contrast, Diverse Depth is able to predict depth more accurately in various scenes, including indoor, outdoor, natural landscapes, and streetscape, etc. Note that, for visualization, we map the output of depth estimation network to a color image. However, unlike the RGB depth map showed in Figure 5, in our experiment, the depth map has only one channel and is normalized before being fed into the network.

## 4. Experiment

In this section, we evaluate our proposed method on the ADE20k dataset. The ADE20k dataset and the experimental configuration are described first. Then, the quantitative and qualitative results are presented. Finally, we present an ablation study on our proposed method.

### 4.1. Dataset and Experiment Configuration

#### 4.1.1. Dataset

The ADE20k dataset contains 20,210 images for training and 2000 images for validation, all of which are high quality pixel-level finely annotated scene images. There are totally 150 semantic categories included for evaluation, including various stuff like sky, road, grass, and discrete objects like personss, cars, and beds [22]. Compared to other semantic segmentation datasets, the ADE20k dataset covers more diverse scene types and object categories, which presents a greater challenge to the segmentation performance of networks. In this paper, we use pixel accuracy and mean IoU (Intersection over Union) to evaluate the results:

(2) $$Pixel\;Acc.=\sum_{i}n_{ii}/\sum_{i}t_{i}$$

(3) $$mean\;IoU=\left(1/n_{c}\right)\sum_{i}\left(n_{ii}/\left(t_{i}+\sum_{j}n_{ji}-n_{ii}\right)\right)$$

where $n_{ij}$ is the number of pixels that is labeled as class *i* and predicted as class *j*, $n_{c}$ indicates the number of classes, and $t_{i}=\sum_{j}n_{ij}$ denotes the number of pixels with ground truth class *i*.

#### 4.1.2. Experiment Configuration

The network is implemented under the pytorch framework. In the training, we use four NVIDIA Tesla V100 (Santa Clara, CA, USA) with 16 GB of GPU memory, and each GPU calculates two images. During training, we fix the parameters of the depth estimation network and only optimize the parameters of the semantic segmentation network (the two encoders and the decoder). We use the cross entropy as the loss function and the SGD optimizer with the base learning rate of 0.02, the momentum of 0.9, and the weight decay of 0.0005. The poly learning rate policy with the power of 0.9 is used for dropping the learning rate. All of the models are trained for 100K iterations with the batch size of 8 on four GPUs. For the backbone networks in our models, we initialize the parameters of the pre-trained model on ImageNet, and the remaining parameters are initialized by Kaiming initialization [85]. In the training, the short side length of the image is randomly resized to one of (300, 375, 450, 525, 600), and flipped randomly. The maximum size of the long side length of the image is limited to 1000. For the initialization of the parameter of the models, we set the random seed of pytorch as 304 in all of our experiments. In addition, during the inference, we adopt a multi-scale testing strategy that averages the output of various input image sizes as our prediction result.

### 4.2. Experimental Results

In the experiment, we provide several optional structures as the encoders. We use DilatedResNet50, DilatedResNet101, and DilatedMobileNetV2 proposed in [22] and HRNetV2 [14] as our RGB encoder separately. For depth encoder, we remove the full connection layers and softmax layers in the original VGG16 [35] and Resnet50 [82], and only retained the full convolution part as our depth encoders. We combine these structures in the manner described in Section 3: when the feature size of RGB encoder is halved, we perform a feature fusion operation with the corresponding RGB features and depth features. All decoders are the aforementioned Pyramid Pooling Module (PPM) [81] structure. Taking the original network structure without depth encoder, we train a total of 12 different network models on the ADE20k dataset, and then evaluate them on the validation set. During training, fusion proportion λ is set to 0.4. Model performance on the ADE20k validation set is shown in Table 1.

From Table 1, we can see that, after the depth encoders are added, the models show certain improvement on the ADE20K validation set. Resnet50 has a better performance than VGG16 as a depth encoder. For mean IoU, Resnet50 has improved by 0.7–1.2% compared with the original network (without depth encoder). VGG16 also has improved by 0.4–0.6%. Among them, the depth encoder improved DilatedResNet50 most, VGG16 and Resnet50 improved 0.6% and 1.2%, respectively as the depth encoder. In addition, the HRNetv2+Resnet50 model achieves the best performance which gets an 82.01% pixel accuracy and 43.98% mean IoU separately. The result indicates that the depth feature can provide a certain amount of information for segmentation, which is consistent with our thinking.

**Figure 6:**
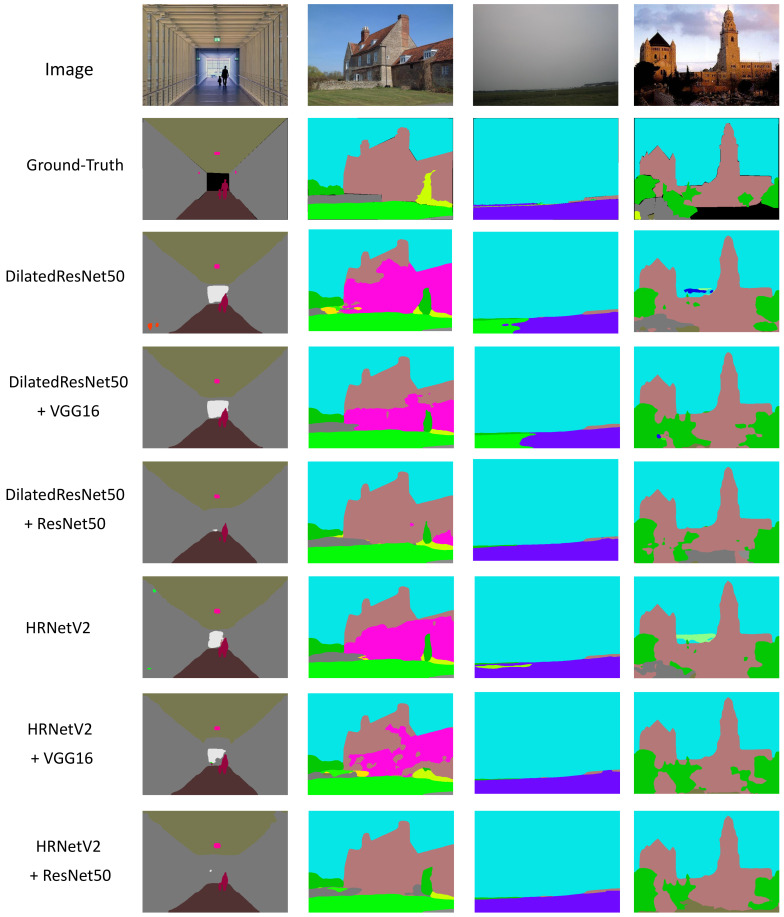
Ground-truth and qualitative results of proposed models on the ADE20k validation set. In the predictions of the second column, several models misclassify the brown class into magenta class. The brown class denotes the “building”, and the magenta class denotes “house”. These two classes are similar, so it is easy to lead to misclassification.

We select some images on the ADE20k validation set and present qualitative results of several models. As can be seen from Figure 6, our proposed models can segment objects more accurately than a basic network (without a depth encoder). In addition, Resne50 performs better than VGG16 as a depth encoder, which is consistent with the performance in Table 1.

Nevertheless, our method inevitably results in an increase in computation. Taking DilatedResNet50 as an example, when the depth encoder is None/VGG16/Resnet50, the average inference time is 29 ms/40 ms/47 ms respectively for a single 480 × 480 RGB image.

Table 2 shows the comparison result of our models and other state-of-the-art methods on the ADE20k validation set. As we can see in the table, the proposed model HRNetV2+Resnet50 performs better on pixel accuracy and mean IoU than other methods. The performance of DilatedResnet50+Resnet50 and DilatedResnet101+Resnet50 are also close to that of existing methods. The poor performance of model DilatedMobileneV2t+Resnet50 is due to the limited performance of base network DilatedMobilenetV2 (as in Table 1), but the performance is still improved compared with the base network. However, we have to admit that our approach has limited advantages over some methods listed in Table 2. However, it is worth noting that our model improves w.r.t the baseline models (>1% Mean IoU on average in Table 1 for Resnet50 depth decoder), and from the qualitative results show by Figure 6, we can see our method improve the performance indeed.

### 4.3. Ablation Study

This section discusses the impact of different network configurations on model segmentation performance. Specifically, we compare our fusion strategy with FuseNet [15]. Then, we evaluate the effect of the fused ratio coefficient λ and the decoder structure. Lastly, we discuss the multi-scale testing strategy and segmentation performance of the model on each class of objects.

#### 4.3.1. Fusion Approach

Our fusion approach is different from FuseNet [15] that uses an element-wise summation for RGB feature and depth feature without any processing. We use the 1 × 1 convolution layers and fused ratio coefficient λ to fuse two kinds of features. We compare our fusion strategy with the method proposed by FuseNet [15] under the same experiment configuration. Specifically, we retrain the FuseNet and our DilatedResnet50+Resnet50 model with the fusion strategy proposed by FuseNet Note that DilatedResnet50 and Resnet50 have the same number of channels of each block, so two kinds of features can be fused without 1 × 1 convolution layers which ensure the feasibility of fusion strategy proposed by FuseNet. As we can see in Table 3, the retrained FuseNet only achieves 71.69% pixel accuracy and 27.81% Mean IoU on ADE20k, which is worse than our models. Considering that the poor performance may be caused by the different backbone architecture, we use the fusion strategy proposed by FuseNet to train our DilatedResnet50+Resnet50 model which gets 79.46% pixel accuracy and 41.62% Mean IoU on ADE20k. The same network architecture that adopts our fusion strategy improves 2.06% and 1.82% on pixel accuracy and Mean IoU, respectively, compared with the fusion strategy proposed by FuseNet.

#### 4.3.2. Fused Ratio Coefficient

In order to explore the influence of fused ratio coefficient λ on segmentation performance, different λ are used for training based on DilatedResNet50+Resnet50+PPM. The results are shown in Table 4.

λ controls the fusion ratio of depth and RGB information. As can be seen from Table 2, when λ = 0.4, the model performs best; when λ is too small, depth information takes up a small proportion in the fused features, and the performance improvement is not obvious. Considering the extreme case λ = 0, the model degrades into the original RGB encoder–decoder model. When the λ is too large, the RGB component is so small in the fusion feature that the model performance is even worse than the original network. Therefore, selecting an appropriate value of λ is crucial for network performance. After extensive attempts, we find that the network performs best when λ = 0.4.

#### 4.3.3. Decoder

**Figure 7:**
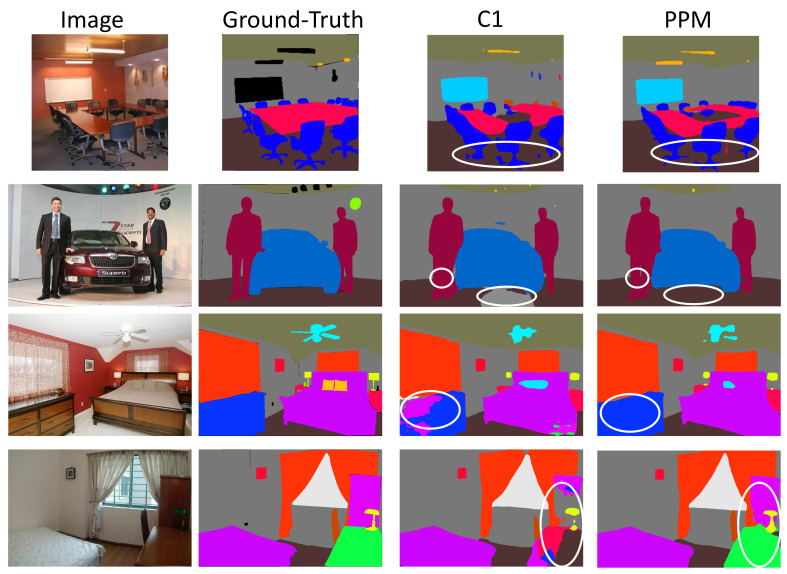
Segmentation results of DilatedResnet50+Resnet50+C1 and DilatedResnet50+Resnet50+ PPM. In the figure, C1 denotes that the decoder only consists of one convolution layer, and PPM denotes the decoder is a Pyramid Pooling Module. From left to right are: input images, ground-truth, the segmentation result of DilatedResnet50+ Resnet50+C1, and the segmentation result of DilatedResnet50+Resnet50+PPM. As can be seen from the part circled by the white circle in the figure, PPM can segment the object more completely, and the segmentation performance of object boundary is also better.

In order to investigate if the PPM decoder works as mentioned above, we remove the multi-scale pooling layer and 1 × 1 convolution layers in PPM which are used to capture the global contextual information of different scales. In other words, the new decoder consists of only one convolution layer and a bilinear interpolation layer. In addition, the new decoder is denoted by C1. We train the DilatedResnet50+Resnet50+PPM and DilatedResnet50+Resnet50+C1 models, and get the results shown in Figure 7.

As shown in Figure 7, we compare the segmentation results of two different networks. Because there is no multi-scale global context information, model DilatedResnet50+Resnet50+C1 cannot completely segment some objects in the scenes (the parts circled by white circles). In contrast, model DilatedResnet50+Resnet50+PPM processes the features using the global average pooling layer of four scales, and effectively obtains the global context information from the input image. In addition, the larger receptive field enables the model to better understand the interrelationships of objects in the scene, which brings about better segmentation performance.

#### 4.3.4. Multi-Scale Testing

The size of the input image has a significant impact on the performance of the segmentation model. In fact, multi-scale is one of the most useful techniques for improving accuracy. In the basic network, feature maps are usually tens of times smaller than the original images, which makes the feature description of small objects difficult to be captured by the network. By introducing multi-scale testing strategy, we can get more robust results than single scale testing. Table 5 presents the comparison results of our models with/without the multi-scale testing strategy. From the table, we can see that the multi-scale testing strategy improves the performance markedly.

#### 4.3.5. Performance on Each Category of Objects

**Figure 8:**
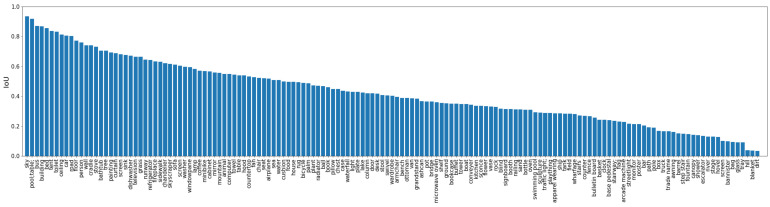
Detailed IoU on the 150 categories given by DilatedResNet50+Resnet50+PPM model. The best segmented categories are big objects, and the worst segmented categories are objects that are usually small and have few pixels.

In order to study the performance of the model on each category of objects, we plot the IoU performance on all the 150 categories which is given by the DilatedResNet50+Resnet50+PPM model, as shown in Figure 8.

As in Figure 8, the model has the best segmentation performance on the sky, buildings, buses, and other large objects, while small objects, such as blanket, tray, and glass, have the worst segmentation performance. This may be caused by large objects taking up more pixels in the image while small objects take up fewer.

## 5. Conclusions

We propose a method of extracting depth information via depth estimation to promote semantic segmentation performance on RGB images. By adding a depth encoder branch to the encoder–decoder network structure, the depth information is gradually fused into the RGB feature and enhance the segmentation performance of the model. We have tried a variety of model structures, and the experimental results show that the proposed method can effectively improve the performance of the original encoder–decoder model.

Future work will focus on trying to improve the performance of depth estimation through the semantic segmentation results. If we get more accurate depth information, it will promote the performance of semantic segmentation. In addition, then we can build a joint multi-task model of depth estimation and semantic segmentation.

## Figures and Tables

**Table 1:** Experimental results of our proposed models on ADE20k validation set. In the Depth encoder column, ‘None’ denotes no depth encoder in the model.

RGB Encoder	Depth Encoder	Pixel Acc. (%)	Mean IoU (%)
Dilated-MobileNetV2	None	78.26	36.28
	VGG16	78.54 (+0.28)	36.79 (+0.51)
	Resnet50	**78.86** **(+0.60)**	**37.31** **(+1.03)**
Dilated-ResNet50	None	80.13	42.14
	VGG16	80.66 (+0.53)	42.75 (+0.61)
	Resnet50	**81.52** **(+1.39)**	**43.40** **(+1.26)**
Dilated-ResNet101	None	80.91	42.53
	VGG16	80.96 (+0.05)	42.96 (+0.43)
	Resnet50	**81.56** **(+0.65)**	**43.54** **(+1.01)**
HRNetV2	None	81.47	43.20
	VGG16	81.64 (+0.17)	43.66 (+0.46)
	Resnet50	**82.01** **(+0.54)**	**43.98** **(+0.78)**

**Table 2:** Comparison of ADE20k validation set. (For simplicity, we only show the result of models whose depth encoder is Resnet50.)

Model	Pixel Acc. (%)	Mean IoU (%)
FCN-8s [34]	71.32	29.39
SegNet [12]	71.00	21.64
DilatedNet [86]	73.55	32.31
RefineNet(resnet152) [87]	79.32	40.70
UperNet(resnet101) [88]	81.01	42.66
HRNetV2 [14]	81.20	43.20
DSSPN(resnet101) [89]	81.39	43.68
PSANet(resnet101) [90]	81.45	43.77
DilatedMobilenetV2+Resnet50	78.86	37.31
DilatedResnet50+Resnet50	81.52	43.40
DilatedResnet101+Resnet50	81.56	43.54
HRNetV2+Resnet50	**82.01**	**43.98**

**Table 3:** Comparison of the fusion strategies of FuseNet [15] and us.

Models *	Pixel Acc. (%)	Mean IoU (%)
FuseNet	71.69	27.81
DilatedResnet50+Resnet50 (fusion strategy of FuseNet)	79.46	41.62
DilatedResnet50+Resnet50 (our fusion strategy)	81.52	43.40

* FuseNet is the original model proposed in [15], DilatedResnet50+Resnet50 (fusion strategy of FuseNet) and (our fusion strategy) denote our DilatedResnet50+Resnet50 model with the fusion strategy proposed by FuseNet and us, respectively.

**Table 4:** The performance of our model using different λ.

λ	0.2	0.4	0.6	0.8
Pixel Acc. (%)	80.22	**81.52**	81.26	79.77
Mean IoU (%)	42.13	**43.40**	43.05	40.34

**Table 5:** The comparison results of our models with/without multi-scale testing strategy. MS denotes the Multi-scale Testing.

Model	MS	Pixel Acc. (%)	Mean IoU (%)
Dilated-MobileNetV2+VGG16+PPM	No	77.69	35.76
	Yes	78.54	36.79
Dilated-Resnet50+VGG16+PPM	No	79.78	41.93
	Yes	80.66	42.75
Dilated-Resnet101+VGG16+PPM	No	80.12	41.87
	Yes	80.96	42.96
Dilated-HRNetV2+VGG16+PPM	No	80.87	42.54
	Yes	81.64	43.66
Dilated-MobileNetV2+Resnet50+PPM	No	77.87	36.78
	Yes	78.86	37.31
Dilated-Resnet50+Resnet50+PPM	No	80.46	42.90
	Yes	81.52	43.40
Dilated-Resnet101+Resnet50+PPM	No	80.43	42.52
	Yes	81.56	43.54
HRNetV2+Resnet50+PPM	No	81.62	43.46
	Yes	82.01	43.98

## Data Availability

Not applicable.
